# Cannabis combined with oxycodone for pain relief in fibromyalgia pain: a randomized clinical self-titration trial with focus on adverse events

**DOI:** 10.3389/fpain.2024.1497111

**Published:** 2024-11-25

**Authors:** Cornelis Jan van Dam, Cornelis Kramers, Arnt Schellekens, Marcel Bouvy, Eveline van Dorp, Mikael A. Kowal, Erik Olofsen, Albert Dahan, Marieke Niesters, Monique van Velzen

**Affiliations:** ^1^ Tackling and Preventing the Opioid Epidemic (TAPTOE) Consortium, Utrecht, Netherlands; ^2^ Department of Anesthesiology, Leiden University Medical Center, Leiden, Netherlands; ^3^ Department of Clinical Pharmacy, Canisius-Wilhelmina Hospital, Nijmegen, Netherlands; ^4^ Department of Pharmacy and Internal Medicine, Radboud University Medical Center, Nijmegen, Netherlands; ^5^ Department of Psychiatry, Radboud University Medical Center, Nijmegen, Netherlands; ^6^ Donders Center for Medical Neuroscience, Donders Institute for Brain, Cognition and Behavior, Nijmegen, Netherlands; ^7^ Nijmegen Institute for Scientist-Practitioners in Addiction (NISPA), Nijmegen, Netherlands; ^8^ Division of Pharmacoepidemiology and Clinical Pharmacology, Utrecht Institute for Pharmaceutical Sciences, University of Utrecht, Utrecht, Netherlands; ^9^ Bedrocan International BV, Veendam, Netherlands; ^10^ Leiden University, Leiden, Netherlands; ^11^ Outcomes Research Consortium, Houston, TX, United States; ^12^ Centre for Human Drug Research, Leiden, Netherlands

**Keywords:** cannabis, oxycodone, self-titration, analgesia, adverse (side) effects

## Abstract

**Objectives:**

We determined whether adding cannabis to oxycodone for chronic non-cancer pain management could reduce treatment-related adverse effects (AEs) while maintaining effective analgesia.

**Methods:**

In this open-label study, fibromyalgia patients aged ≥18 years were randomized to receive 5 mg oxycodone tablets (max. four times/day), 150 mg of inhaled cannabis containing 6.3% Δ^9^-tetrahydrocannabinol and 8% cannabidiol (max. times inhalation sessions/day), or a combination of both for 6 weeks. The primary endpoint was treatment-related adverse events, assessed using a 10-point composite adverse event (cAE) score; additionally, we recorded daily reported pain relief and daily tablet and cannabis consumption.

**Results:**

In total, 23 patients were treated with oxycodone, 29 with cannabis, and 29 with the oxycodone/cannabis combination. Three patients from the oxycodone group (13%) and 18 patients from the cannabis groups (31%, 9 in each group) withdrew from the trial within 2–3 weeks because of the severity of AEs. There were no differences in treatment-related cAE scores among the three groups that completed the study (*p* = 0.70). The analgesic responder rate showed a ≥1- point reduction in pain in 50% and a ≥2-point reduction in 20% of patients, while 50% of patients experienced no treatment benefit. The combination treatment reduced oxycodone tablet consumption by 35% (*p* = 0.02), but it did not affect the number of cannabis inhalation sessions.

**Conclusions:**

Cannabis combined with oxycodone offered no advantage over either treatment alone, except for a reduction in opioid tablet intake; however, the overall drug load was the highest in the combination group. Moreover, cannabis was poorly tolerated and led to treatment discontinuation in one-third of participants treated with cannabis.

**Clinical Trial Registration:**

The trial was registered at the WHO International Clinical Trials Registry Platform (trialsearch.who.int) on July 26, 2019, identifier NL7902.

## Introduction

Opioids are widely prescribed for the management of chronic non-cancer pain, despite the awareness that they offer only temporary pain relief, are associated with an increase in dose due to tolerance development, come with a multitude of side effects (including dizziness, sedation, nausea/vomiting, constipation, hyperalgesia, addiction, and respiratory depression), and pose challenges when attempting to discontinue them due to the development of dependence and withdrawal symptoms (1, 2). One way to reduce or even eliminate opioid consumption is to manage pain with a multimodal approach that includes pharmaceutical-grade cannabis (3). We have previously shown that a single dose of inhaled pharmaceutical-grade cannabis variety, specifically Bediol that contains Δ^9^-tetrahydrocannabinol (THC) and cannabidiol (CBD), can reduce experimental pain and spontaneous pain in patients with fibromyalgia syndrome for short durations (4). Prior research indicates that using several cannabis varieties (all non-pharmaceutical-grade varieties) for the management of chronic pain may lead to a reduction in opioid consumption, albeit not in all patients (5–8). However, a systematic review from 2020 that included more than 7,000 non-cancer patients with chronic pain found that while opioid dosages decreased in the majority of patients when medicinal cannabis was combined with opioids, all included studies had a high risk of bias and causal inference was often not possible (9). At present, there is no consensus on whether combining opioids with cannabis yields a positive health benefit, i.e., an improved balance between pain relief and adverse effects (AEs); additionally, the optimal cannabis doses for reducing opioid consumption remain unknown (9).

In our current study, we hypothesized that adding the cannabis strain Bediol to oxycodone treatment would have advantages over oxycodone or cannabis alone in treating patients with fibromyalgia experiencing moderate-to-severe pain. The study was designed to allow patients to self-titrate their oxycodone and cannabis doses (with strict limitations) over a 6-week period (10). The primary objective of the study was to assess and compare drug-related side effects in fibromyalgia patients treated with either a combination of cannabis and oxycodone vs. oxycodone or cannabis alone. At this point, we would like to highlight that opioids and cannabinoids are not recommended for the treatment of fibromyalgia, but some patients do receive these treatments, highlighting the need to study their side effect profiles and develop strategies for weaning off these drugs. For further guidance, see also the recommendations of EULAR, NICE, and the Health Council of the Netherlands (Gezondheidsraad) on chronic pain treatment in fibromyalgia (11–13).

## Methods

### Ethics and registration

The Medical Ethical Review Board of Leiden University Medical Center approved the study protocol, which was subsequently published (10). All participants provided written informed consent prior to enrollment in the study. The study followed the Consolidated Standards of Reporting Trials (CONSORT) reporting guidelines and was performed from December 2019 to November 2022. The trial was registered on the WHO International Clinical Trials Registry Platform (trialsearch.who.int) on 26 July 2019, with the identifier NL7902.

### Patients

Patients aged 18 years or older, of either sex, with a diagnosis of fibromyalgia, were recruited. Inclusion criteria included chronic (>3 months) pain with a pain score of ≥5 (on a scale from 0 = no pain to 10 = most severe pain imaginable) for most of the day and a diagnosis of fibromyalgia according to the 2016 American College of Rheumatology criteria (4, 14, 15). These criteria include a widespread pain index of ≥7 (on a scale from 0 to 19) and a symptom severity score of ≥5 (on a scale from 0 to 12), or a widespread pain index of 3–6 with a symptom severity score of ≥9. The widespread pain index defines the number of body areas where a patient experienced pain during the previous week; the symptom severity score indicates the level of other core symptoms of fibromyalgia, such as fatigue, non-refreshing sleep, and cognitive issues (14).

Exclusion criteria included (10) the presence of a medical condition or comedication that could alter the pharmacokinetics of cannabis or oxycodone, allergy to the study medication, prior use of cannabis, inability to taper previous pain medications (including strong opioids or tramadol) within 2 weeks prior to dosing, history of illicit drug abuse or alcohol abuse, history of psychosis, pregnancy or lactation, and the presence of pain syndromes other than fibromyalgia. The use of any opioid other than the oxycodone provided by investigators during the trial was not allowed. f patients had used opioids before participating in the study, the drug was tapered to zero within the 2 weeks prior to dosing. The use of paracetamol, gabapentin, or pregabalin was allowed, provided these medications had been consumed at a fixed dose for at least 6 weeks. The presence of autonomic symptoms, such as diarrhea, constipation, or dizziness, was not a reason for exclusion, as these are consistent with fibromyalgia syndrome (14, 15).

### Study design and treatment

The study followed an open-label, randomized design. Patients with chronic pain from fibromyalgia were randomized in a 1:1:1 ratio to self-titrate 5 mg oral oxycodone sustained-release tablets (Mundipharma Pharmaceuticals BV, The Netherlands), 150 mg of inhaled cannabis (Bediol, Bedrocan International BV, The Netherlands) containing 6.3% THC (9.5 mg) and 8% CBD (12 mg) with a specific terpene profile (including myrcene, α_2_-pinene, terpinolene, β-caryophyllene, cis-ocimene, α-terpineol, all >0.5 mg/g; see https://bedrocan.com/wp-content/uploads/bediol-terpene-profile.pdf) or their combination for 6 weeks with a 6-week follow-up. Patients who were treated with cannabis received capsules that contained 150 mg of cannabis of the Bediol variety. These capsules could be placed in a hand-held vaporizer, the Mighty Medic (Storz & Bickel GmbH & Co., Germany), which was provided to patients for home use. The vaporizer heats the plant material to allow the conversion of THC and CBD acids into active THC and CBD for inhalation. The patients inhaled cooled cannabis vapor from the vaporizer in sessions lasting 3–5 min. The study was performed at a single center (LUMC) where patients received their medication; however, all patients were treated at home. The cannabis formulation and dosage used in this study were based on our earlier study comparing the effects of three cannabis varieties, which found that the Bediol variety was most effective in reducing fibromyalgia pain (4).

Randomization was performed by the study team using the Castor data capture system (www.castoredc.com) on the day prior to dosing. Patients assigned to oxycodone treatment could use up to two (5 mg) oxycodone tablets per day during week 1 of treatment, increasing to up to four oxycodone tablets per day during weeks 2–6. Patients assigned to cannabis treatment were instructed by the study team to use up to three (150 mg) cannabis capsules per day in week 1, increasing to up to five capsules in weeks 2–6. Patients assigned to the combination of cannabis and oxycodone were allowed to use up to two oxycodone tablets and up to three cannabis capsules per day in week 1, increasing to up to four oxycodone tablets and up to five cannabis capsules per day during treatment weeks 2–6. Medication was prepared by the pharmacy and provided to patients through the study team.

### Measurements

#### Endpoints

The primary study outcome was the number of daily AEs reported during the course of treatment. For this purpose, a practical cAE score was used, which included the following 10 symptoms: dizziness (when getting up), sleepiness, insomnia, headache, nausea, vomiting, constipation, drug high, hallucinations, and paranoia. These symptoms encompassed both opioid and cannabis AEs. The participants were requested to score these symptoms at the end of each treatment day as they had occurred during that day. Scoring of each symptom resulted in 1 point (minimum score per day = 0, indicating no symptoms; maximum score per day = 10, indicating all symptoms occurred; maximum score for the entire 42-day treatment period = 420). The comparisons were the cAE scores observed in the oxycodone/cannabis arm vs. either treatment alone. While the severity of AEs was not considered in the cAE scores, it was considered when patients decided to withdraw from the study. In such cases, the occurrence of AEs was assessed quantitatively (moderate, severe, and unacceptable).

The secondary endpoint of the study was the average pain experienced over the previous 24 h, specifically related to fibromyalgia, which was scored daily by the patients. The pain was scored on an 11-point numerical rating scale (NRS) from 0 (no pain) to 10 (worst pain imaginable). We opted for this simpler NRS score over the more complex scoring used during screening, as we believed it to be more reliable.

Finally, the number of oxycodone tablets taken and the number of cannabis inhalation sessions were considered exploratory endpoints.

### Data analysis

#### Sample size

Sample size calculations were based on the primary outcome, the cAE score for AEs occurring from drug treatment. To this end, a power analysis was performed using Monte Carlo simulations. Briefly, the side effect data were assumed to be binomially distributed. In total, 100 simulated data sets were fitted to a mixed effects model (using the glmer function in R), and the number of times the *p*-values for the significance test of the interaction term (time × treatment) fell below alpha = 0.05 was counted. In the opioid-only group, the AE counts were assumed to be approximately 5 over time, while in the cannabis/oxycodone group, the counts were assumed to decrease by 20% over time to 4. The inter-occasion and interindividual variability of the time × treatment effect was assumed to have an SD of 0.3 at the end of the 6-week period. For a power of 80%, 22 participants were needed in each arm; for a power of 90%, 29 participants were needed. The 20% decrease in AE counts was based on consensus within our pain groups and prior study experiences, where a 20% decrease in AEs was deemed a significant improvement in quality of life by the patients (4, 10).

#### Statistical analysis

We performed intention-to-treat and per-protocol analyses on cAE data and pain scores, along with a per-protocol analysis on tablet usage. cAE and pain data were compared across treatments using linear mixed models in the NONMEM statistical package version 7.5.1 (ICON Development Solution, USA); *p*-values were computed using the *χ*^2^-test based on differences of minus two log-likelihood values. Missing data were imputed using linear interpolation. cAE and pain scores were analyzed from days 14 to 42 of treatment. We excluded weeks 1 and 2 from the cAE and pain relief analyses, as these weeks were considered the titration phase leading to a steady-state treatment phase; however, they were considered when reporting dropouts. Dropouts were analyzed using Kaplan–Meier analysis and the log-rank (Mantel–Cox) test in GraphPad Prism, version 10.2.3 for Mac OS (GraphPad Software, Boston, MA, USA). Reported medication use was compared between groups using unpaired non-parametric *t*-tests in GraphPad Prim 10. *p*-values <0.05 were considered significant. Participants who dropped out of the study were replaced to ensure that 20 participants completed the study in each treatment arm; all patients who started the study were included in the intention-to-treat analysis (10).

## Results

**Figure 1:**
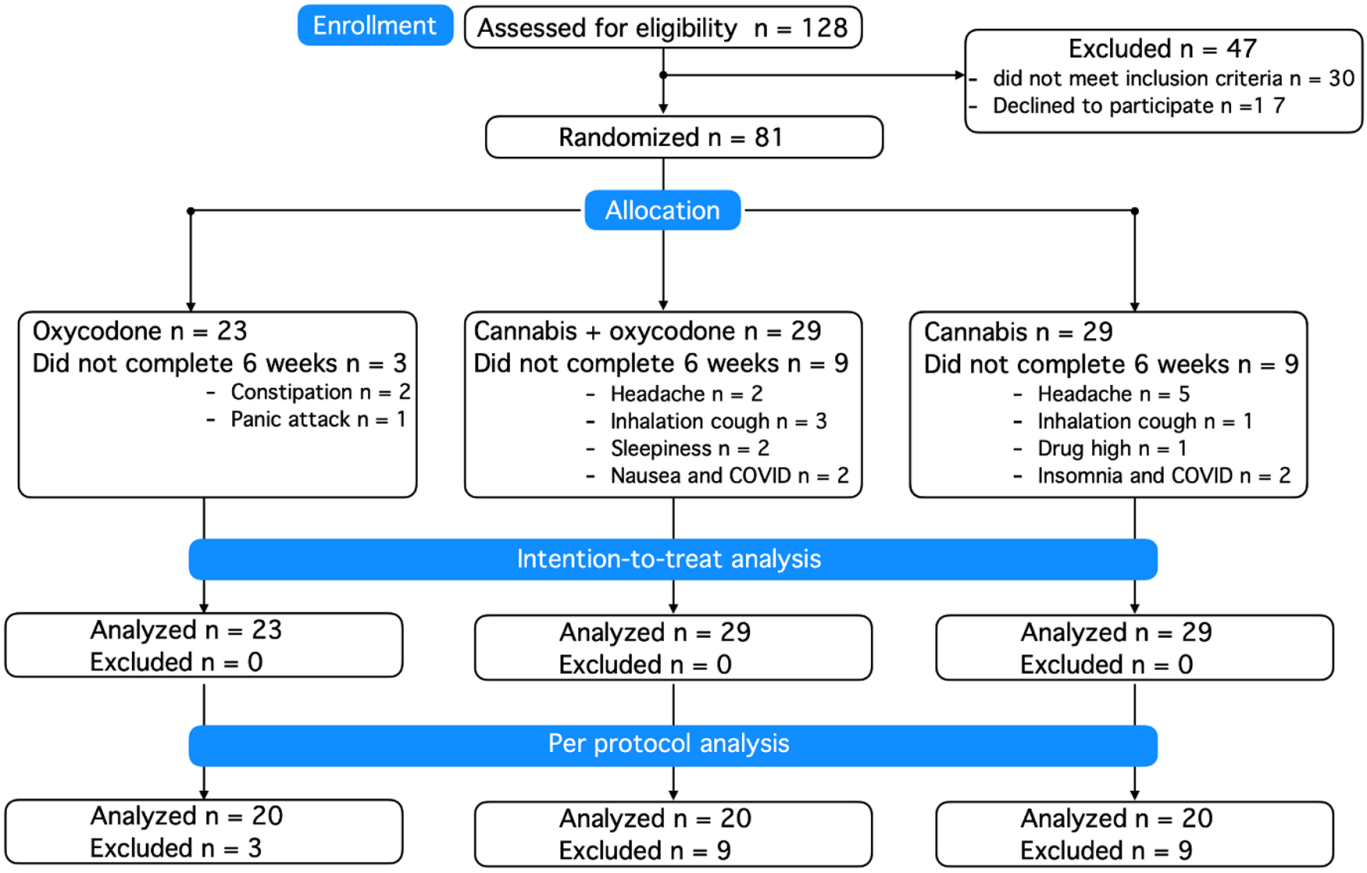
Consort flow diagram.

A total of 128 patients were screened for eligibility (Figure 1). Patient characteristics at baseline are given in Table 1, showing little difference between groups. The majority of included patients were women, with just two men included in the study. Dropouts did not differ in demographic or disease characteristics from those who successfully completed the 6-week treatment period. Prior to their inclusion in the study, 20% of randomized patients used no analgesic medication, 34% used one analgesic, and 46% used at least two analgesics (median 2, range 2–4). There was no difference in analgesic use between treatment groups, with the most frequently used drugs being paracetamol (42% of patients), non-steroidal anti-inflammatory drugs (42%), opioids (tramadol, codeine, oxycodone: 22%), amitriptyline (16%), pregabalin (12%), and duloxetine (6%).

**Table 1:** Patient characteristics at baseline.

	Oxycodone	Cannabis + oxycodone	Cannabis
Total number of patients	23	29	29
Women	23	28	28
Age (years)	46.9 ± 16.0	37.1 ± 13.4	44.2 ± 14.1
Weight (kg)	77.5 ± 17.1	76.0 ± 13.3	70.8 ± 13.3
Height (cm)	168 ± 6	169 ± 8	167 ± 7
BMI (kg/m^2^)	27.1 ± 5.7	27.2 ± 4.3	27.1 ± 5.2
Fibromyalgia symptoms			
Widespread pain index (0–19)	14.3 ± 3.1	14.5 ± 2.9	15.1 ± 3.1
Symptom severity (0–12)	8.6 ± 1.7	8.3 ± 1.7	8.7 ± 2.1
Tender points (0–18)	13.8 ± 3.9	13.6 ± 3.1	14.3 ± 2.6
Pain characteristics			
Average score	6.3 ± 1.0	6.6 ± 1.1	6.4 ± 1.1
Maximum pain	7.9 ± 1.0	8.1 ± 1.1	7.7 ± 1.4
Patient history, *n*			
Musculoskeletal	23	29	29
Gastrointestinal	7	9	3
Respiratory	6	3	6
Cardiovascular	5	0	1
Neurologic	1	3	3
Immunologic	3	4	3
Genitourinary	1	6	1
Mental health, *n*			
Depression	13	12	15
Post-traumatic stress disorder	5	9	4
Panic disorder	8	9	3
Phobia disorder	6	9	9

All data are mean ± SD unless otherwise stated.

**Figure 2:**
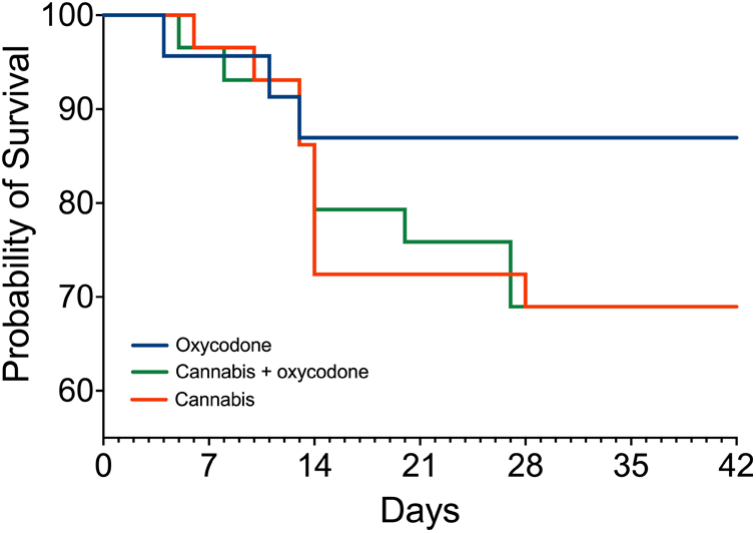
Survival curves: number of patients in the study. Blue line: oxycodone group; orange line: cannabis group; green line: combined cannabis and oxycodone group. In the oxycodone group, 23 patients began treatment, while 3 patients dropped out. In the other two groups, 29 patients started treatment, while 9 patients dropped out.

A total of 81 patients were randomized and treated, of whom 21 (26% of treatment-exposed participants) decided to end their consent to participate in the first 2–3 weeks of treatment due to the occurrence of AEs; see the Kaplan–Meier curve (Figure 2). In total, 18 participants treated with cannabis (9 in each group, 31%) dropped out of the study because of what we consider cannabis-related AEs, such as headache, coughing upon inhalation, drug high, sleepiness, or insomnia. In agreement with the protocol, all participants who dropped out were replaced to ensure that 20 patients in each treatment group successfully completed the trial. Dropout rates did not differ between treatment groups (log-rank Mantel–Cox test, *p* = 0.08), but we argue that they approached significance given the small sample sizes per group, favoring oxycodone over treatments including cannabis.

### Composite adverse event score

#### Intention-to-treat analysis

**Figure 3:**
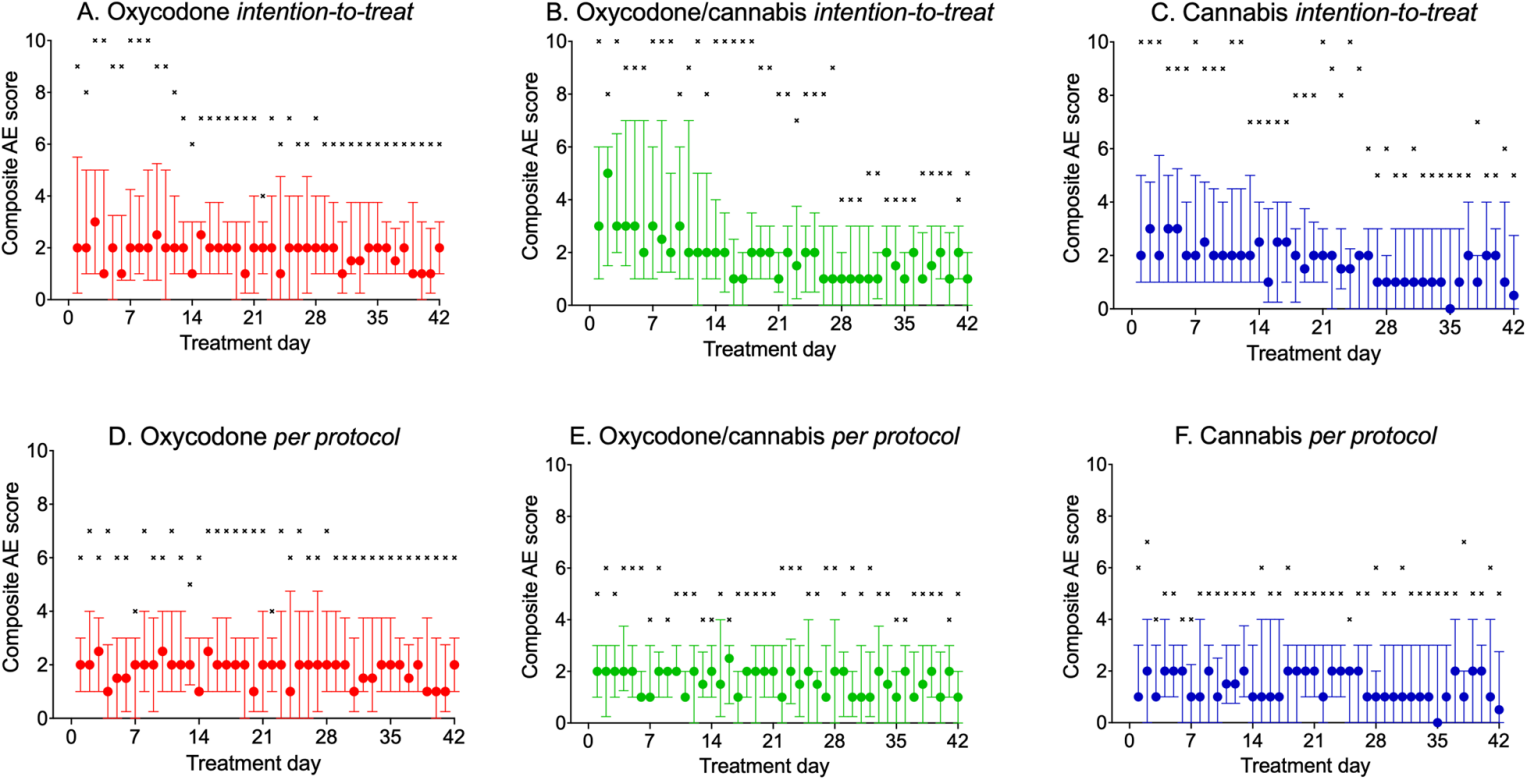
(**A–F**) Daily reported composite adverse event score during the 42 days of treatment. **(A–C)** Intention-to-treat population; (**D–F**) per-protocol treatment. Circles: median ± interquartile ranges; ×: maximum reported pain scores.

cAE scores are shown in Figure 3A–C. The graphs indicate that the number of cAE scores was higher in the first 2 weeks of treatment in the cannabis and cannabis/oxycodone groups, with particularly high maximum cAE scores (8–10) and elevated median scores. In the first 2 weeks of treatment, median AE scores ranged from 1 to 3 in the oxycodone, 2–5 in the oxycodone/cannabis, and 2–3 in the cannabis groups. From day 14 onward, median cAE scores over time were 2 (oxycodone), 1.5 (oxycodone/cannabis), and 1.5 (cannabis), with no difference in cAE scores among the three treatment arms (linear mixed model: *p* = 0.97).

#### Per-protocol analysis

The results of the analyses are shown in Figures 3D–F, and indicate that the 60 patients who completed the study had median cAE scores ranging from 1 to 2 throughout the study period. Total cAE scores were 91 ± 64 (mean ± SD) for oxycodone, 72 ± 53 for cannabis, and 76 ± 43 for the oxycodone/cannabis combination. Mixed model analysis revealed that the scores over time were not different between treatment arms (*p* = 0.70). The different components of the cAE score contributed equally to the score, with somewhat less constipation in the cannabis group and more reports of drug high in the combination group. The most prevalent scores were for sleepiness, headache, insomnia, and constipation (occurring in >10% of patients; Table 2). Paranoia and hallucinations were rarely reported by the participants and contributed <0.2% to the cAE; vomiting contributed 1% to the cAE. All patients tapered their treatment at the end of the 6-week period, and no additional AEs related to withdrawal were reported by the patients.

**Table 2:** Components of the composite adverse event score and distribution of the 60 patients who completed the protocol across treatment groups.

Component of score	Oxycodone	Oxycodone + cannabis	Cannabis
Dizziness	12	12	8
Sleepiness	24	20	20
Insomnia	16	23	17
Headache	14	23	21
Nausea	14	7	10
Vomiting	1	1	1
Constipation	15	8	13
Drug high	4	6	10
Hallucinations	0	0	0
Paranoia	0	0	0
Total	100	100	100

Data are percentages of the total score for that specific treatment.

### Pain relief

**Figure 4:**
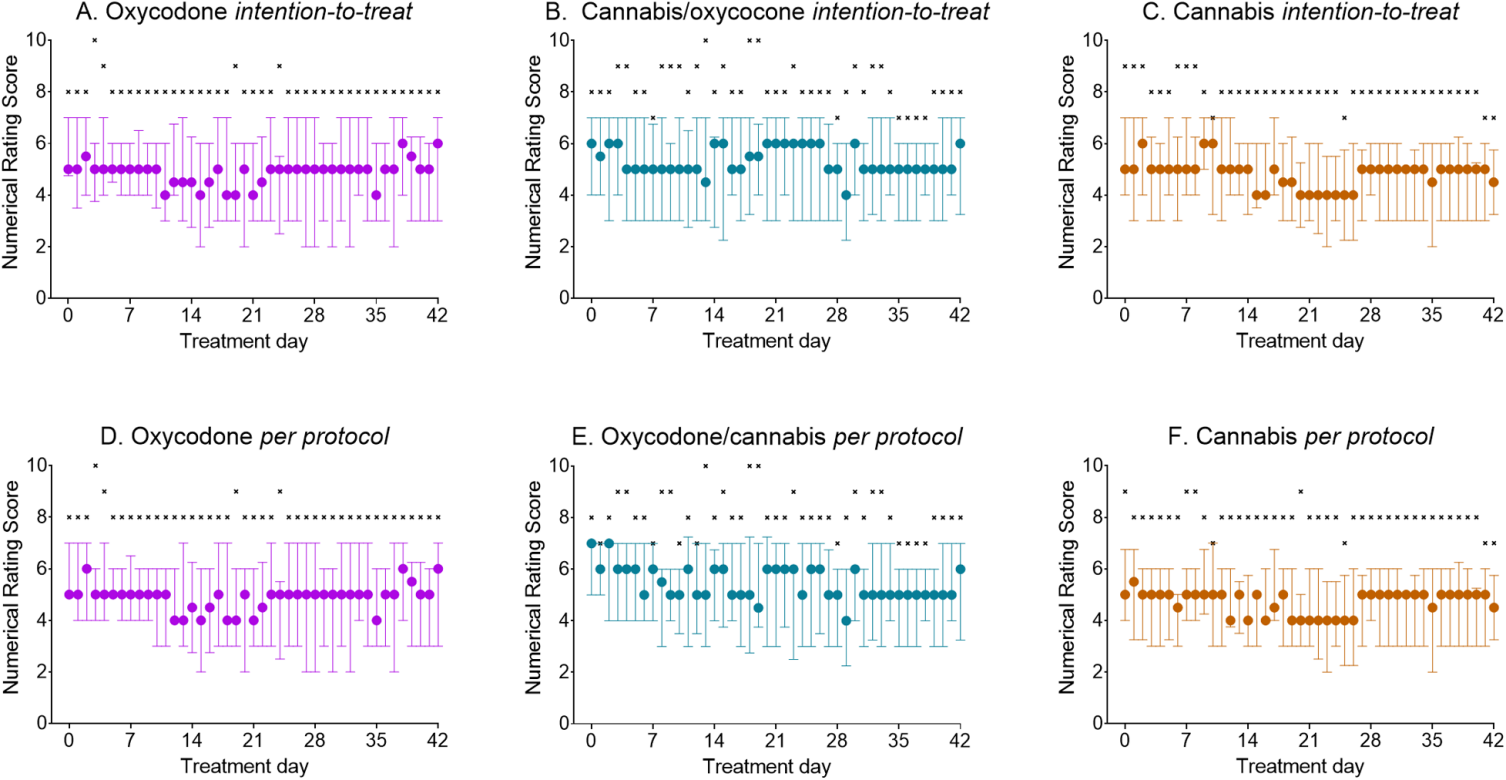
(**A–F**) Daily reported pain scores during the 42 days of treatment. **(A–C)** Intention-to-treat population; **(D–F)** per-protocol treatment. Circles: median ± interquartile ranges; ×: maximum reported pain scores.

Median pain scores in the intention-to-treat and per-protocol groups were not different between treatment arms (both analyses, *p* > 0.05; Figure 4). Pain scores showed little change over time across all three treatment arms. The per-protocol data analysis showed that irrespective of treatment, approximately 50% of patients experienced a decrease of 1 or more points on the NRS and 20% experienced 2 or more points. Approximately 25%–30% of patients showed no clinically relevant analgesic benefit (NRS change < 1) from any treatment, while 20%–35% of patients experienced an increase in pain scores over time (median change +0.6 NRS points, interquartile range 0.3–1.1 NRS points). There were no differences in the responder rate distribution between treatments.

### Medication use

**Figure 5:**
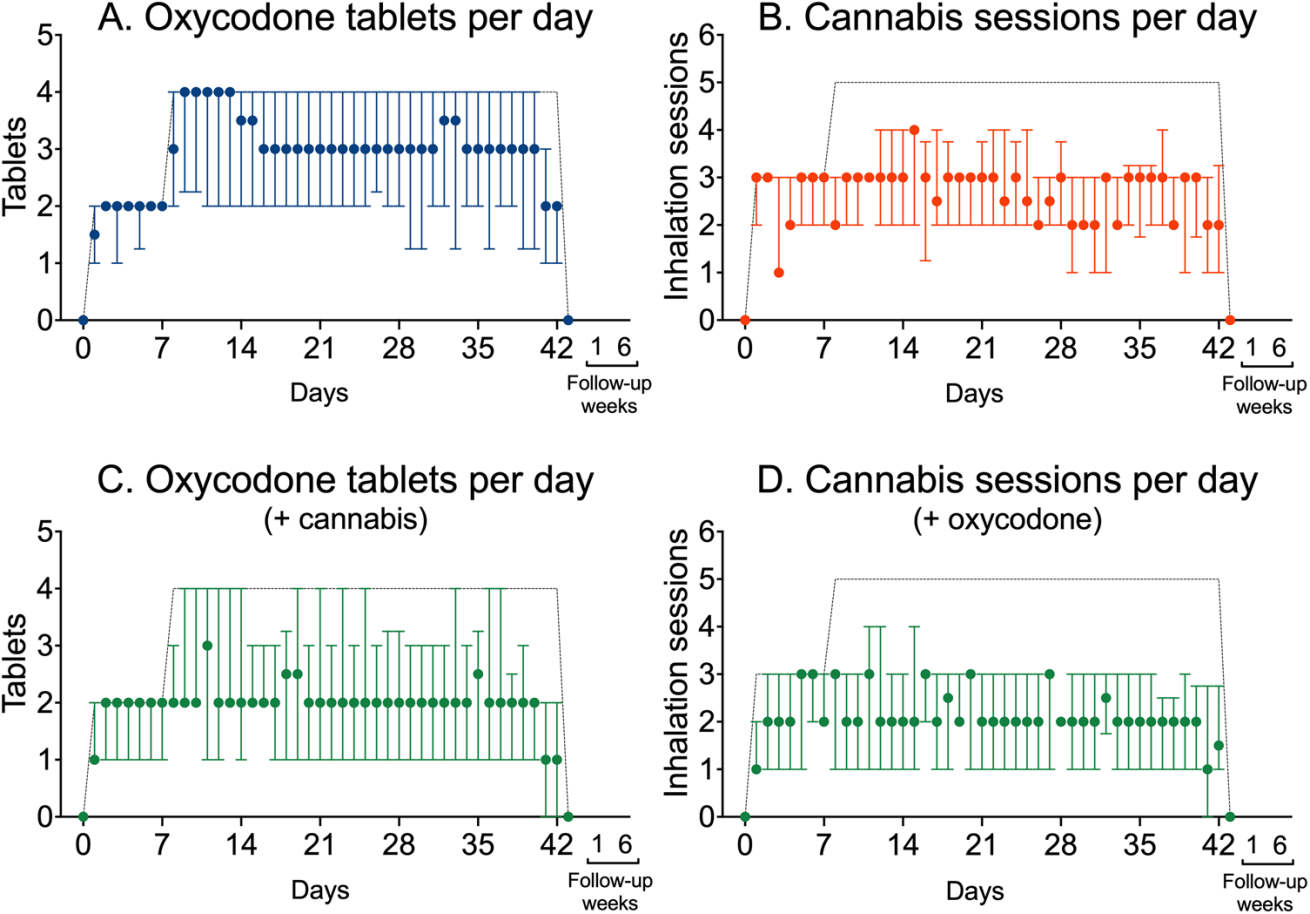
Use of oxycodone tablets and cannabis inhalation sessions in the three treatment groups. **(A)** Median oxycodone tablets (±interquartile ranges) used per day in patients who received oxycodone only. **(B)** Median number of inhalation sessions per day (±interquartile ranges) in patients who received cannabis only. **(C)** Median oxycodone tablets (±interquartile ranges) used per day in patients that additionally received cannabis. **(D)** Median number of inhalation sessions per day (±interquartile ranges) in patients who additionally received oxycodone. The dashed lines depict the maximum allowed oxycodone doses and cannabis inhalation sessions.

The mean ± SD total number of oxycodone tablets consumed during the trial was 112 ± 37 or 3 tablets/day (median with range 1–4) in the oxycodone group compared to 83 ± 40 in the combined oxycodone/cannabis group (2 tablets per day with range 0–3), or a difference of 28 tablets in the study (95% CI 3–53), which reflects a median difference of 1 tablet per day (with 95% CI 0.2–1.5, *p* = 0.02). The mean number of total cannabis inhalation sessions was 101 ± 37 (median 2 inhalation sessions per day with range 1–4) in the cannabis arm compared to 86 ± 35 inhalation sessions (median 2 inhalation sessions per day with range 1–4) in the combined oxycodone/cannabis arm, with a difference of 16 inhalation sessions in the study (95% CI −7 to 39), which reflects a median difference of 0 inhalation sessions per day (with 95% CI −1 to 0.2, *p* = 0.23). The total drug load in the combined arm was 169 (cannabis + oxycodone = 83 + 86). Figure 5 shows the medium number of tablets and inhalation sessions per group, depicting the up- and down-titration of oxycodone and cannabis across the three treatment groups. The difference in drug use between single and combined treatments (A vs. C, and B vs. D) was approximately one oxycodone dose and one cannabis inhalation session per day. It also shows that tapering occurred successfully in the last days before the end of the study.

## Discussion

The main outcomes of our study in fibromyalgia patients treated with cannabis combined with oxycodone or oxycodone or cannabis alone in a self-titration paradigm are as follows: (i) approximately one in four participants dropped out of the study, mainly due to the severity of AEs experienced from inhaled cannabis, irrespective of the treatment group; (ii) there were no significant differences in pain scores between treatments, with 30% of patients experiencing ≥1 NRS (but <2 NRS) pain reduction, 20% experiencing ≥2 NRS, and 50% reporting no analgesic benefit from any treatment; (iii) the drug load increased in patients treated with the combination of oxycodone and cannabis relative to either treatment alone; and (iv) patients titrated their dosing based on AEs and pain relief, an indication that patients dosed themselves according to the utility of treatment (where utility is defined as the maximization of the difference between analgesic effects and adverse effects) (16). Our findings challenge the belief that combining cannabis with an opioid reduces opioid consumption with fewer AEs. Nonetheless, our data suggest that cannabis may benefit a small subset of patients who were able to endure the inhalation of cannabis and reported a reduction in pain scores of at least one point.

### Adverse effects

The finding that 18 patients discontinued treatment (Figure 2) indicates that a significant proportion of our patients were not well-suited for pain management with cannabis. Not only did they experience a high number of daily AEs, but the severity of symptoms was compelling enough to discontinue treatment within the first weeks. Headaches were particularly severe and occurred in 12% of dropouts (Table 2). Despite the high number of dropouts, at this point, we cannot exclude that there may possibly be a role for cannabis monotherapy in chronic pain management because of its effectiveness and a low number of AEs, however, in just a minority of patients. Since prediction of treatment outcomes is challenging, more research is needed to identify *a priori* which patients may benefit the most from cannabis. In addition, we acknowledge that there is a risk of cannabis use disorder, and consequently, cannabis treatment should be applied with careful monitoring.

Our suggestion that combining oxycodone with cannabis would reduce the number of side effects compared to either treatment alone was not supported. This outcome may be attributed to the self-titration of the study medication, which allows patients to adjust their medication use when side effects occur too frequently or are more intense than acceptable. These findings challenge the traditional treatment paradigm that prescribes fixed daily doses and instead support a more flexible or adaptive dosing strategy, particularly for drugs like cannabis and opioids that are known for their wide range of AEs.

### Pain relief and drug load

The analgesic effect of the oxycodone–cannabis combination was comparable to that of either treatment alone (Figure 4). This outcome was achieved with less oxycodone consumption (26% reduction) without significantly affecting the number of cannabis inhalation sessions. Although the reduced intake of oxycodone tablets is an important and relevant observation that is often pursued in clinical practice, the total drug load (cannabis + oxycodone) exceeded that of either treatment alone. Given the high potential for misuse associated with these two drug classes and the absence of additional benefit from their combination, we argue that our study does not simply support the concurrent use of cannabis and oxycodone for the management of chronic pain. Possibly, in patients who use oxycodone, cannabis could be added to slowly taper opioid use. However, further studies are needed to explore this approach.

### Comparison with the literature

In line with our findings, various studies reported a high incidence of AEs from cannabis for chronic pain treatment (17, 18). In contrast, a 2024 review on opioids and cannabis in chronic pain found both treatments to be moderately effective for pain relief, with cannabis causing fewer dropouts than opioids (19). This discrepancy with our study may be related to the use of different cannabis varieties or differences in patients’ experience with cannabis . Regarding the combined use of opioids and cannabis for chronic non-cancer pain, a recent systematic review of retrospective studies and surveys concluded that cannabis significantly reduced self-reported opioid consumption compared to patients who did not use cannabis (9). These findings contrast with ours, which we attribute to the critical bias of the included studies, the wide range of cannabis use (1.5 mg–2 g), and the study populations, which consisted of experienced cannabis users.

### Study limitations

Several study limitations deserve discussion:

(i) Except for the patients who discontinued participation, we remained uninformed on the intensity or severity of subjectively experienced AEs. While this may be considered a caveat, our *a priori* reasoning was that intensity is difficult to score for patients, particularly when multiple symptoms occur concurrently or when symptoms need to be scored just once at the end of the day, complicating the implementation of the study. It is our experience that patients more frequently complain about the occurrence of symptoms rather than about their intensity. Patients who remained in the study deemed the intensity of their symptoms acceptable, while those who dropped out deemed their AEs severe and unacceptable. Moreover, by using the prespecified cAE, we may have missed some adverse events.
(ii) The high number of dropouts among individuals who received cannabis introduces a potential bias, particularly when assessing subjective symptoms and pain relief. The absence of difference between the *per-protocol* and intention-to-treat analyses suggests a minimal influence of dropouts on the study outcomes. This was further substantiated by a sensitivity analysis that showed that, with the number of dropouts in our study, the power to detect differences between study arms had decreased to approximately 80%. In addition, the high number of dropouts indicates that cannabis may be an unwise choice for some patients and that alternative treatments should be probed.
(iii) We designed a cAE score based on fixed symptoms. While we allowed the reporting of other symptoms, we may have missed some that were not part of the current scoring system. The cAE score comprised symptoms that we expected to occur with opioid use (e.g., constipation, nausea), cannabis use (e.g., drug high, hallucinations, paranoia), or both treatments (dizziness, sleepiness). Interestingly, the distribution of side effects did not seem to diverge between treatments.
(iv) Adding a placebo control could have provided a clearer baseline for evaluating the effectiveness of combination therapy. Although the absence of a placebo group may have affected the outcome of our study, we argue that comparing three distinct treatments in a similar fashion likely distributed any placebo (or nocebo) effects across all three treatment groups. In addition, adding a placebo is difficult in psychedelic drug research, as full blinding appears to be difficult, and factors such as patient expectations may affect study outcomes. This may possibly be reduced by using active placebos (20).
(v) The study population was predominantly female, which reflects the sex distribution of the disease. However, this limits the generalizability of our study beyond the female population and beyond fibromyalgia.
(vi) We excluded patients with a history of cannabis consumption to standardize our study population. Therefore, this further limits the extrapolation of our study results to the broader chronic pain population. Possibly, patients with prior experience with cannabis consumption would have had fewer adverse events compared to our cannabis-naïve population. This may have overestimated the effect of cannabis on adverse outcomes and the dropout rate in our current population.
(vii) cAE and pain scores were analyzed from days 14 to 42 of treatment. To assess how this affected our study outcome, we performed *post-hoc* analyses of the complete 52 days of the study. Although this did not affect the outcome, the large number of dropouts in all three study arms during this period made us decide to exclude these study days from the main analyses.

## Conclusions

In this relatively small open-label trial involving patients with chronic pain from fibromyalgia, which is best considered exploratory, we showed that self-titration with oxycodone, cannabis, or their combination was successfully completed by 75% of the study population. However, 25% of patients found the AEs intolerable and discontinued participation in the first 2–3 weeks of the trial. Importantly, patients treated with cannabis dropped out at three times the rate (31%) of participants assigned to just oxycodone (12%). Irrespective of treatment, the responder rate of patients who completed the 6-week treatment period did not differ between treatment groups. The combination of oxycodone and cannabis did not lead to fewer AEs or a decrease in total drug load, indicating that this combination may not offer additional benefit and may even carry a greater risk of harm due to the abuse and toxicity potential of these two drugs compared to using only one drug. Still, a small subset of patients who did tolerate cannabis self-titration achieved an acceptable efficacy with limited side effects (median cAE score 2). More research with larger sample sizes, inclusion of placebo groups, and proper characterization of specific patient phenotypes is needed to predict tolerance to cannabis therapy and its therapeutic effectiveness for the management of chronic pain. For now, we would argue that the use of cannabis is best restricted as a last-resort option for patients with therapy-resistant chronic pain, with careful monitoring of therapeutic effects and AEs. Finally, we would like to emphasize once more that opioids are not recommended for treating chronic pain in fibromyalgia patients. See treatment guidelines in (11–13).

## Data Availability

The raw data supporting the conclusions of this article will be made available by the authors, pending reasonable request and agreement upon the protocol from the corresponding author.
